# Comparative effects of ultrasound, ultraviolet, and microwave treatments on the structure-property relationships of cactus polysaccharide-gelatin composite films

**DOI:** 10.1016/j.ultsonch.2026.108079

**Published:** 2026-09-23

**Authors:** Shikuan Zhao, Muhammad Aaqil, Feng Zhang, Rui Zhou, Siyu Zhou, Yuanfeng Chen, Cheng Gong, Huiqing Luo, Chongye Fang, Cunchao Zhao

**Affiliations:** a College of Food Science and Technology, Yunnan Agricultural University, Kunming 650201, China; b Yunnan Plateau Characteristic Agricultural Industry Research Institute, Yunnan Agricultural University, Kunming 650201, China; c Yunnan International Joint Laboratory of China-Cambodia Signiture Agro-Products Green Development, Kunming 650201, China

**Keywords:** Cactus polysaccharide, Gelatin composite films, Ultrasound, Physical-field treatment, Active packaging

## Abstract

Biodegradable polysaccharide–protein films are promising alternatives to petroleum-based packaging but often lack adequate structural stability and functionality. Although ultrasound (US), ultraviolet (UV), and microwave (MW) treatments have been individually applied to improve biopolymer films, a systematic comparison on the same film system remains unreported. Here, cactus polysaccharide (CP) was extracted from Opuntia spp. with high purity (91.69% polysaccharides, 117.15 μg/g protein, 1.03 mg/g phenolics). CP–gelatin films were fabricated and treated with US (200 W, 20 kHz, 30 min), UV (254 nm, 30 W, 30 min), or MW (300 W, 2450 MHz, intermittent heating). Effects of these physical fields technologies on the structural, physicochemical, mechanical, and functional properties of the films were systematically evaluated. Compared with UV and MW treatments, US treatment resulted in significantly lower moisture content (6.38% vs. 7.09% and 6.78%), water solubility (65.34% vs. 71.51% and 68.22%), swelling index (99.0% vs. 117.7% and 108.5%), and water vapour permeability (1.81 × 10^−7^ vs. 2.44 × 10^−7^ and 2.25 × 10^−7^ g·m^−2^·Pa^−1^·s^−1^), along with the highest tensile strength (20.53 vs. 16.91 and 14.38 MPa), antioxidant activity (ABTS: 93.80% vs. 91.93% and 92.37%; DPPH: 82.48% vs. 78.29% and 81.88%), and UV-shielding performance (UV-A: 85.07% vs. 71.72% and 75.40%; UV-B: 96.99% vs. 91.37% and 93.47%). UV increased visible-light transparency but showed the lowest UV-blocking efficiency, while MW induced localized crystallization and the highest surface hydrophobicity (WCA: 87.56° vs. 74.52° for US and 76.95° for UV). All treatments slowed soil biodegradation versus control. Overall, US provided the most balanced enhancement of structural integrity, barrier performance, antioxidant activity, and UV protection, highlighting its potential for sustainable active packaging applications.

## Graphical abstract

**Figure:**
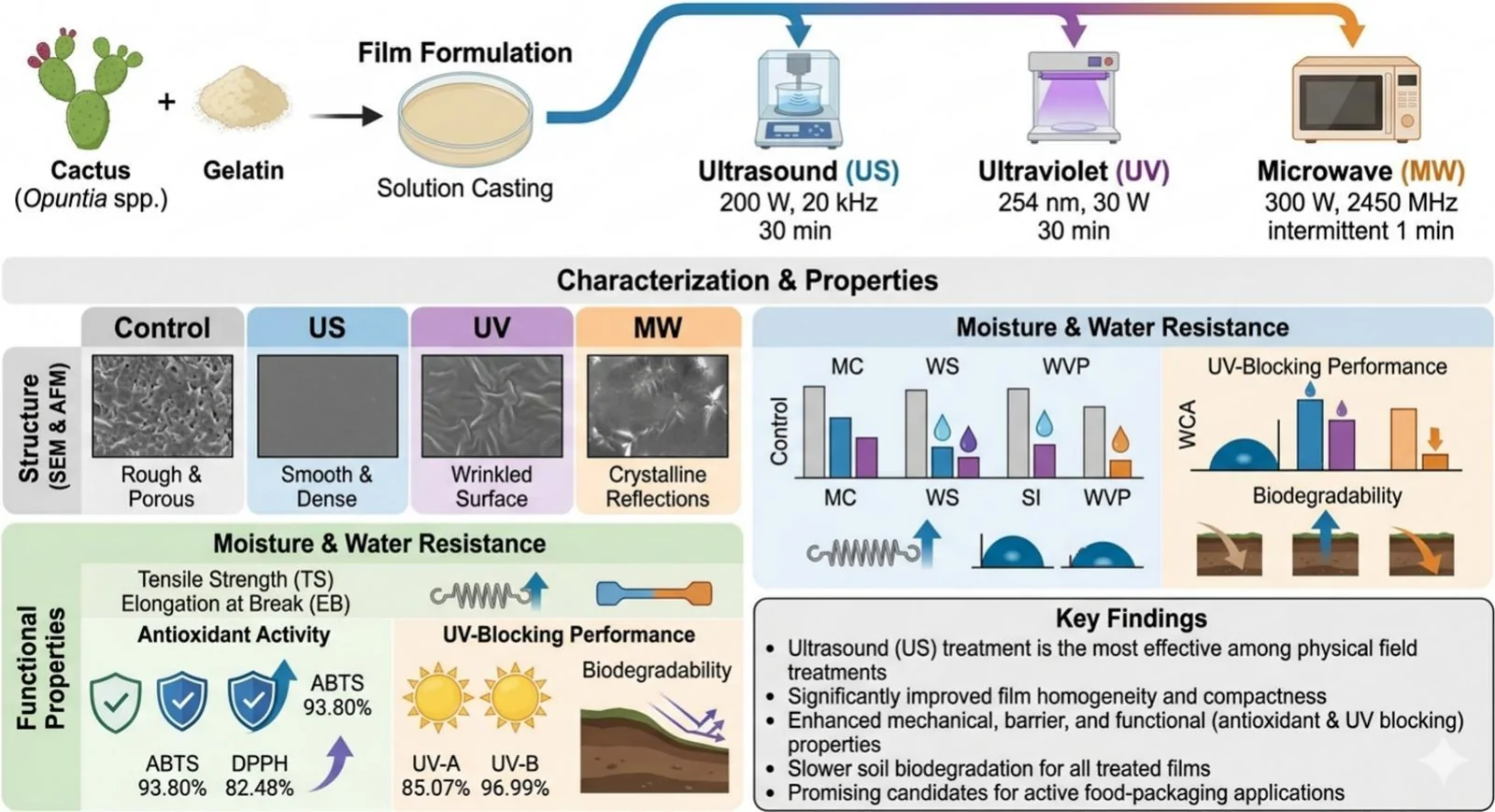

## 1 Introduction

The escalating environmental concerns associated with non-biodegradable petroleum-based packaging materials have accelerated the development of edible and biodegradable films derived from natural biopolymers [1]. This crisis has driven substantial research toward sustainable alternatives that can reduce dependence on fossil–based plastics while maintaining food preservation functions. Among these materials, gelatin has emerged as an attractive film-forming biopolymer because of its excellent film-forming ability, strong oxygen-barrier properties, and wide commercial availability (Reji, Mathew, V, Sabu, & Roy, 2025). However, gelatin-based films are highly sensitive to moisture and often exhibit insufficient mechanical strength, arises from gelatin's hydrophilic nature and its tendency to form brittle films without adequate structural reinforcement. These shortcomings limit their practical application in food packaging.

Blending gelatin with polysaccharides represents an effective strategy to overcome these limitations, as polysaccharides can reinforce the polymer network through hydrogen bonding and electrostatic interactions [59]. In this context, cactus polysaccharide (CP), extracted from *Opuntia* spp., has attracted considerable interest owing to its high water-holding capacity, gelling properties, and inherent antioxidant and antimicrobial activities. The prickly pear cactus is abundant in arid and semi-arid regions, making CP a renewable and low-cost biopolymer resource with promising potential for food packaging applications [29], [48]. Nevertheless, like many biopolymer blends, CP–gelatin composite films often require further structural and functional enhancement to meet the diverse demands of practical food packaging, particularly regarding barrier properties and mechanical performance. In this context, post-processing physical-field treatments offer a green and effective means to tailor film properties without altering the primary chemical structure or introducing synthetic additives.

Physical-field technologies, including ultrasound, ultraviolet (UV) irradiation, and microwave treatment, have recently gained attention as environmentally friendly and efficient approaches for modifying the properties of biopolymer films [45]. Ultrasound generates acoustic cavitation, which disrupts molecular aggregates and promotes the uniform dispersion of film components, thereby enhancing film compactness and barrier properties. UV irradiation induces photo-crosslinking of protein chains, improving structural stability and surface characteristics. Microwave treatment provides rapid volumetric heating that can alter polymer-chain mobility and promote localized crystallization [4], [57]. Each technology offers distinct advantages and acts through fundamentally different mechanisms, suggesting their effects on film structure and performance may differ substantially. Although these technologies have been investigated individually in various polysaccharide–protein systems, several critical research gaps remain. First, a systematic, parallel comparison of US, UV, and MW treatments on the same film system has not been performed, making it difficult to rationally select the most appropriate technology for specific requirements. Second, the mechanisms through which each physical field modulates multiscale structure from molecular conformation and crystalline ordering to surface chemistry have not been comprehensively elucidated. Third, the direct correlation between specific structural modifications and functional outcomes remains poorly understood [20] (Scheme 1).

**Scheme 1:**
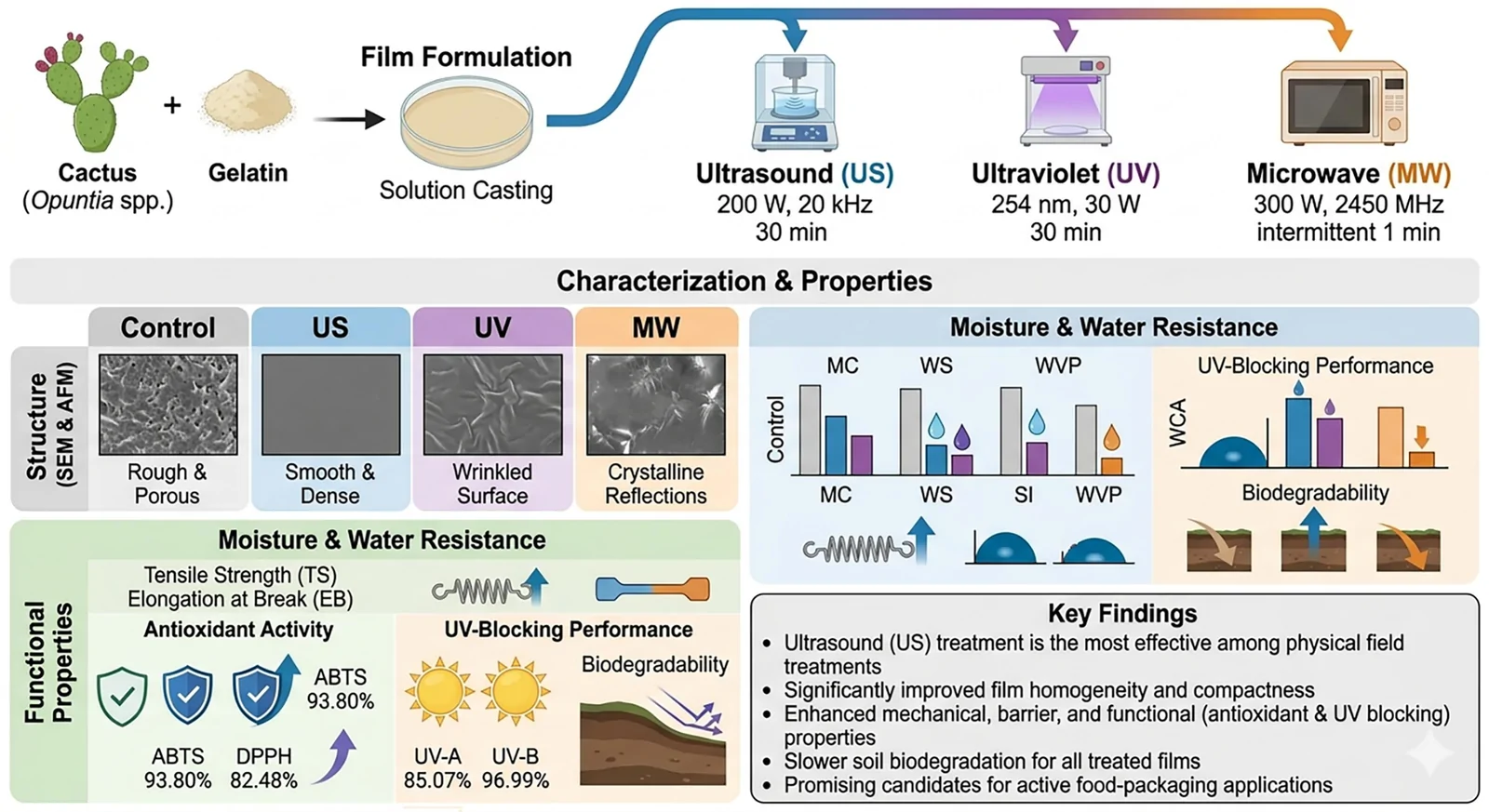
Graphical abstract illustrating the effects of ultrasound (US), ultraviolet (UV), and microwave (MW) treatments on the structure–property relationships and active packaging applications of cactus polysaccharide–gelatin composite films.

Therefore, this study provides a parallel, mechanism-oriented comparison of ultrasound, UV, and microwave treatments on CP-gelatin films under processing conditions carefully selected from literature reports [2], [10], [1]. Rather than claiming absolute superiority of one technology, we aim to elucidate how each physical field distinctly modulates multiscale structure from molecular conformation and crystalline ordering to surface chemistry and how these structural changes translate into specific functional outcomes. To achieve this, a comprehensive range of analytical techniques (SEM, AFM, FTIR, XPS, XRD, TGA, DSC) was employed to characterize film structure and molecular organization, alongside evaluations of thickness, mechanical performance, moisture content, water solubility, swelling behavior, water–vapor permeability, water contact angle, optical properties, antioxidant activity, and soil biodegradability. By providing a systematic comparison of three representative physical-field technologies, this study offers new insights into structure–property relationships in CP–gelatin composite films and identifies an effective strategy for developing high-performance active packaging materials.

## 2 Materials and methods

### 2.1 Materials and reagents

Fresh cactus (Opuntia spp.) was obtained from Guangnan County, Wenshan Prefecture, Yunnan Province, China. Gelatin was purchased from Zhejiang Jiadeli Biotechnology Co., Ltd. (Zhejiang, China), while glycerol was supplied by Beijing Lanjieke Technology Co., Ltd. (Beijing, China). All other reagents were of analytical grade and used without further purification.

### 2.2 Preparation and compositional analysis of cactus polysaccharide

CP was extracted from dried *Opuntia* powder via hot-water extraction, followed by ethanol precipitation, Sevag deproteinization, dialysis, and freeze-drying. The extraction yield was determined gravimetrically. The chemical composition of purified CP was analyzed by determining total polysaccharide content using the phenol–sulfuric acid method, protein content using the Coomassie Brilliant Blue assay, and total phenolic content using the Folin–Ciocalteu method. The more detailed methodology is attached to the supplementary materials section.

### 2.3 Fabrication of multifunctional CP–gelatin films subjected to different physical treatments

The CP–gelatin composite films were prepared using a solution-casting method [27]. Gelatin (2%, w/v) was dissolved in water at 80 °C with glycerol (5 wt%) as a plasticizer, followed by the addition of CP (10 wt% based on the dry weight of gelatin). The film-forming solution was divided into four portions: untreated control, ultrasound (US, 200 W, 20 kHz, 30 min, ice bath), ultraviolet (UV, 254 nm, 30 W, 30 min), and microwave (MW, 300 W, 2450 MHz, intermittent heating for 1 min) treatments. Following treatment, the solutions were cast and dried. Detailed procedures are provided in the Supplementary Material.

### 2.4 Structural characterization of films

Film surface morphology was examined using SEM (LEO 1530 VP) and AFM (Bruker Dimension Icon). FTIR spectra (Nicolet iS20, 4000–500 cm^−1^, resolution 4 cm^−1^) were recorded to investigate molecular interactions. Surface elemental composition was determined by XPS (Thermo Scientific K-Alpha), while crystalline structure was analyzed by XRD (Rigaku Miniflex 600, Cu Kα). The more detailed methodology is attached to the supplementary materials section.

### 2.5 Thermogravimetric analysis and differential scanning calorimetry

Thermal properties were characterized using TGA (Netzsch STA 2500, N_2_ atmosphere, 30–800 °C at 10 °C/min) and DSC (TA Instruments DSC 2500, N_2_ atmosphere, −40 °C to 400 °C at 10 °C/min, modulated mode). Approximately 8 mg and 5 mg of sample were used for TGA and DSC analyses, respectively. Indium, aluminum, and sapphire were employed as calibration standards.

### 2.6 Physicochemical and mechanical properties of the films

Film thickness was measured using a digital micrometer. Mechanical properties, including tensile strength and elongation at break, were determined using a universal testing machine according to ASTM D882 [34]. Moisture content (MC) and water solubility (WS) were determined gravimetrically after drying and re-drying [35]. The swelling index was calculated from weight gain after 12 h of immersion. Water-vapour permeability (WVP) was measured gravimetrically [38], and water contact angle (WCA) was determined using a video contact-angle analyzer [52].

### 2.7 Optical properties

Colour parameters (L*, a*, b*, and ΔE) were measured using a Konica Minolta CR-400 colorimeter with a white reference plate (L* = 97.75, a* = −0.49, b* = 1.96) according to [21]. UV–Vis transmittance in the wavelength range of 200–800 nm was recorded using a spectrophotometer. UV-A and UV-B blocking efficiencies were calculated following [23].

### 2.8 Antioxidant assay

The antioxidant activities of the films were evaluated using ABTS•^+^ and DPPH• radical-scavenging assays according to the method reported by [37]. Film samples (50 mg) were immersed in 10 mL of either 7 mM ABTS solution or 2.45 mM DPPH methanolic solution and incubated in the dark at room temperature for 30 min. Absorbance was subsequently measured at 734 and 517 nm, respectively. Antioxidant activity was calculated using the following equation:

$$\text{Antioxidant activity}\;\left(\text{\%}\right)=\frac{{\text{(A}}_{\text{0}}\text{-}{\text{A}}_{\text{T}}\text{)}}{{\text{A}}_{\text{0}}}$$

where A_0_ = absorbance value for the control and A_T_ = absorbance values for the film samples, respectively.

### 2.9 Evaluation of biodegradability

Film biodegradability in soil was evaluated according to the method described by [51]. Soil was collected from an outdoor site at a depth of 0–15 cm. Dried film specimens were individually placed in soil-filled pots and buried at a depth of 4–5 cm. Soil moisture was maintained throughout the experiment by regular water spraying.

### 2.10 Statistical analysis

All experiments were performed in triplicate, and results are expressed as mean ± standard deviation. Statistical analysis was conducted using one-way analysis of variance (ANOVA) followed by Duncan's multiple-range test in SPSS version 26.0 (IBM, NY, USA). Differences were considered statistically significant at p < 0.05.

## 3 Results and discussion

### 3.1 Compositional analysis of CP

Cactus polysaccharide (CP) was extracted from dried *Opuntia* powder through hot-water extraction, ethanol precipitation, deproteinization, dialysis, and freeze-drying. The extraction yield was 14.22%, indicating a moderate recovery of polysaccharides from the raw plant material [24]. The chemical composition of purified CP was subsequently determined. The total polysaccharide content reached 91.69% (w/w), confirming the high purity of the extract. This result highlights the effectiveness of the Sevag deproteinization and dialysis procedures in removing non-saccharide constituents. The protein content was only 117.15 μg/g, demonstrating efficient removal of protein impurities during purification. In addition, the total phenolic content was 1.03 mg/g, indicating that a small fraction of phenolic compounds remained associated with the polysaccharide matrix or was not completely removed during purification [13]. Overall, the extracted CP exhibited high purity and was therefore suitable for subsequent structural and functional investigations in hydrocolloid-based food applications.

### 3.2 Morphological analysis

The surface morphology of CP–gelatin composite films subjected to different physical treatments was examined by SEM to elucidate structural changes within the biopolymer matrix (Fig. 1A). The untreated film exhibited a relatively rough surface with randomly distributed microaggregates, indicating limited compatibility and localized aggregation between cactus polysaccharide and gelatin under conventional stirring conditions. In contrast, ultrasound treatment generated a more homogeneous, dense, and smooth microstructure. This improvement can be attributed to acoustic cavitation and microstreaming effects, which disrupted molecular aggregates and promoted the uniform dispersion of CP within the gelatin matrix [56]. The US-treated film also exhibited a highly integrated network structure, consistent with the reduced d-spacing observed in the XRD analysis.Fig 2.

**Fig. 1:**
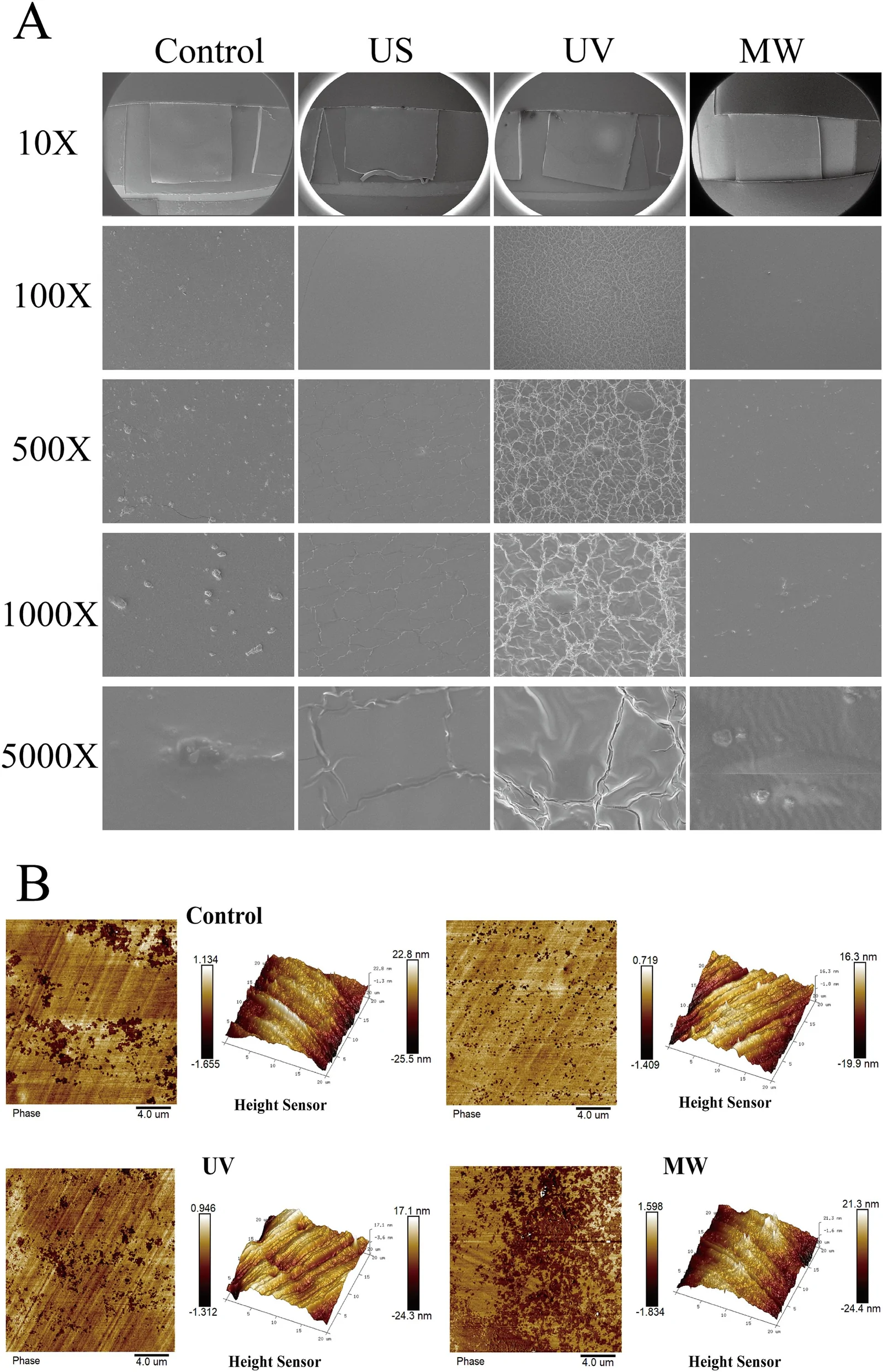
(A) Scanning electron microscopy (SEM) images of cactus polysaccharide–gelatin composite films under different physical treatments. (B) Atomic force microscopy (AFM) height images showing the surface topography of the corresponding films.

**Fig. 2:**
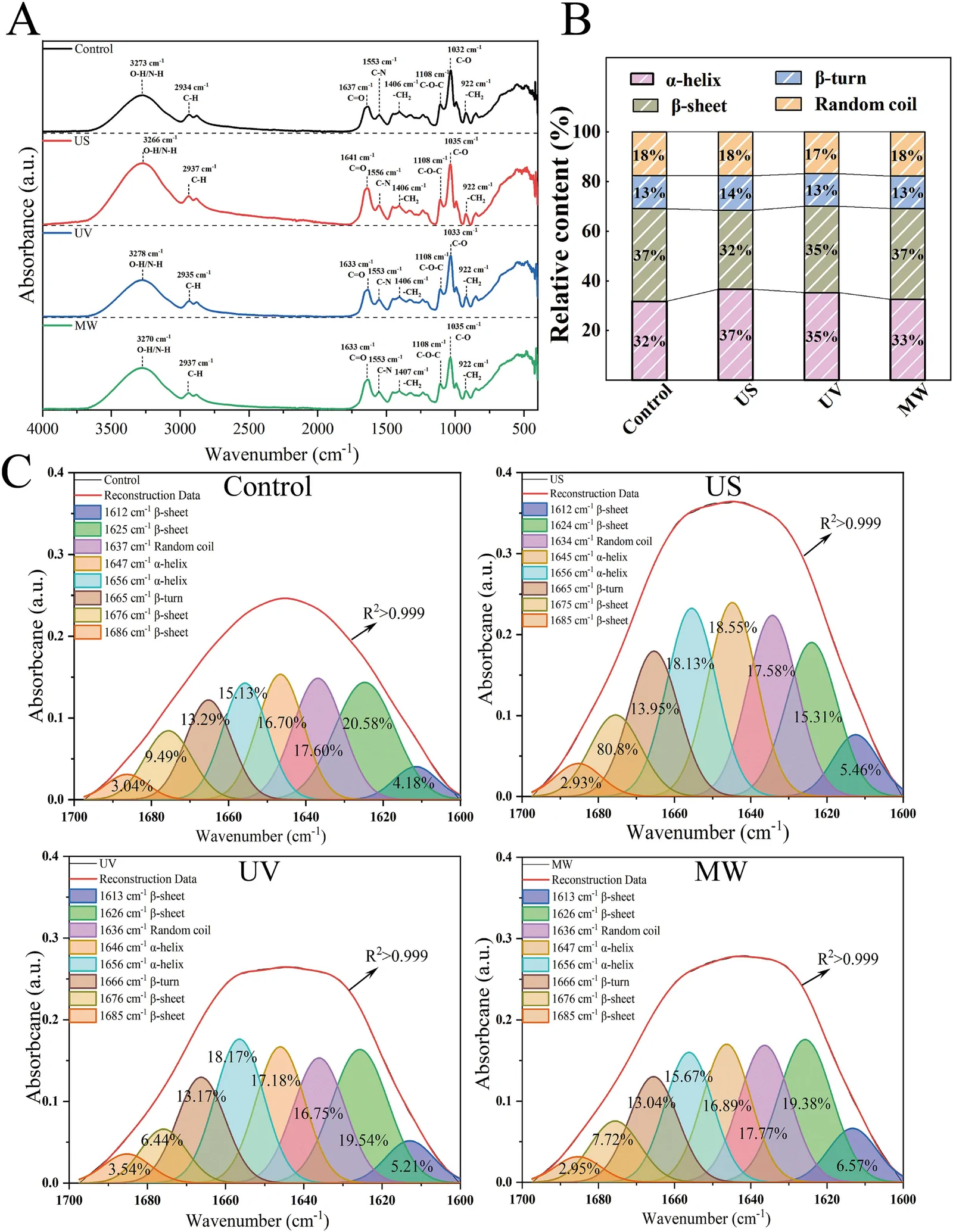
(A) FTIR spectra (4000–400 cm^−1^) of cactus polysaccharide-gelatin composite films under different physical treatments, presented in absorbance mode; (B) Relative contents of protein secondary structures of cactus-gelatin composite films under different treatments; (C) Deconvoluted FTIR spectra in the amide I region (1600–1700 cm^−1^) of different physical treatments.

UV treatment markedly altered the film morphology, producing a compact yet visibly wrinkled and reticulated surface. This structural feature may result from UV-induced photo-crosslinking, which increased crosslink density and generated internal contractile stresses during film formation [18]. In contrast, the MW-treated film displayed a distinctive porous morphology characterized by microcrater-like structures. Rapid volumetric heating and dipole polarization associated with microwave irradiation likely promoted localized microboiling and accelerated water evaporation, thereby generating pores within the solidifying matrix [25]. Collectively, these observations demonstrate that physical-field treatments, particularly ultrasound, effectively modulate interfacial compatibility and structural integrity in CP–gelatin films, thereby contributing to improved barrier performance for active packaging applications.

The surface nanostructure of the films was further characterized by atomic force microscopy (AFM) (Fig. 1B). Peak-to-valley height range and phase contrast were employed as indicators of surface roughness and nanoscale compositional homogeneity, respectively [46]. The control film exhibited a height range of 48.3 nm and a phase contrast value of 2.789, reflecting moderate surface roughness and nanoscale heterogeneity. Following ultrasound treatment, both parameters decreased substantially (height range: 36.2 nm; phase contrast: 2.128), indicating the formation of a smoother and more homogeneous surface through acoustic cavitation [14]. This observation is consistent with the established role of ultrasound in disrupting molecular clusters and promoting the development of cohesive and uniformly structured networks [39]. UV treatment produced intermediate values (height range: 41.4 nm; phase contrast: 2.258), suggesting that photo-crosslinking primarily modified the film surface without substantially affecting roughness or phase separation. Conversely, microwave treatment increased phase contrast to 3.432 while maintaining a relatively high height range (45.7 nm). These findings suggest that microwave-induced thermal effects promoted localized aggregation or microphase separation between gelatin and cactus polysaccharides, resulting in increased nanoscale heterogeneity without markedly reducing surface roughness [15].

The AFM results further support the observed structure–property relationships. The US-treated film exhibited the lowest roughness and phase contrast values, which corresponded to the lowest water-vapour permeability and swelling index. This observation is consistent with previous findings that smoother and more homogeneous surfaces improve barrier performance [32]. In contrast, the MW-treated film exhibited the highest phase contrast, intermediate barrier properties, and the highest water contact angle. As reported by [16], nanoscale heterogeneity can enhance hydrophobicity without severely compromising barrier function. Overall, ultrasound generated the smoothest and most homogeneous surface, whereas microwave treatment introduced nanoscale heterogeneity that may be advantageous for applications requiring enhanced hydrophobicity.

### 3.3 Fourier-transform infrared (FTIR) spectroscopy analysis

Fourier-transform infrared (FTIR) spectroscopy was used to investigate intermolecular interactions and conformational changes in CP–gelatin composite films subjected to different physical treatments. The full-range spectra (Fig. 3A) confirmed that all samples retained the characteristic absorption bands of the biopolymer matrix, indicating that the applied physical treatments primarily influenced secondary intermolecular interactions without altering the primary chemical structure [12]. A detailed analysis of the amide A region (3000–3600 cm^−1^), associated with O–H and N–H stretching vibrations, revealed noticeable band broadening accompanied by significant wavenumber shifts across the treated samples. In particular, ultrasound-treated films exhibited a downward shift in wavenumber, suggesting the formation of a stronger hydrogen-bonding network. To quantitatively assess the hydrogen bonding strength, the peak position of the amide A band was determined for each sample after baseline correction and peak fitting. The amide A band exhibited a downward shift of approximately 7 cm^−1^ in US-treated films compared with the control (from 3273 cm^−1^ to 3266 cm^−1^), indicating a strengthening of intermolecular hydrogen bonding (the magnitude of wavenumber decrease is positively correlated with hydrogen bond strength). This behavior may be attributed to acoustic cavitation, which promotes partial unfolding of gelatin chains and enhances intermolecular interactions between hydroxyl and amino groups within the composite matrix [31].

**Fig. 3:**
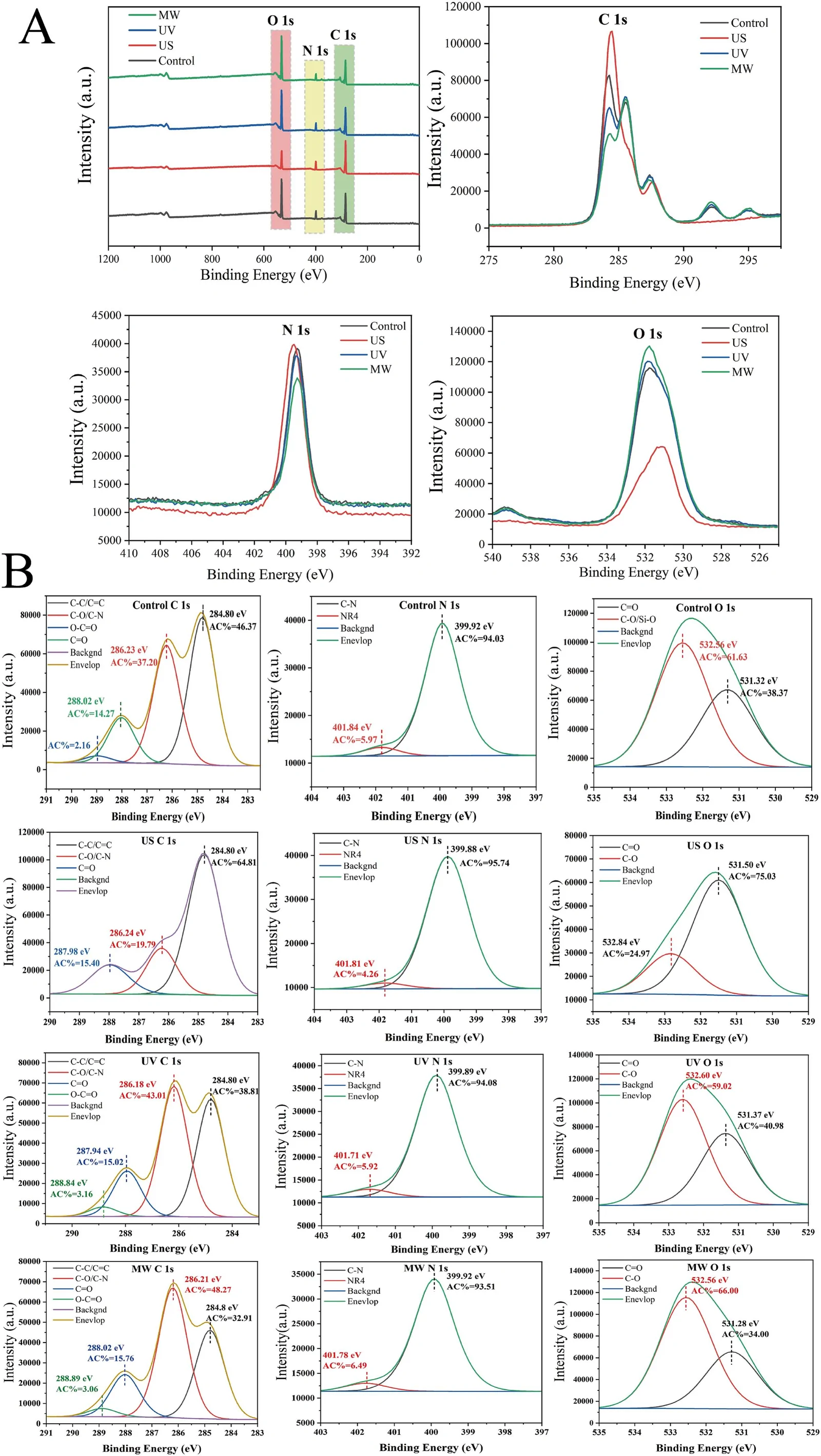
XPS characterization of cactus–gelatin composite films under different physical treatments: (A) full survey spectra, (B) high–resolution deconvoluted C1s, N1s, and O1s core–level spectra.

As shown in Fig. 3B and Table 1, ultrasound treatment significantly increased the α-helix content from 31.82% to 36.68% while reducing the β-sheet content from 37.29% to 31.79%, indicating improved molecular ordering. The ultrasound-induced conformational transition toward a more ordered α-helix-rich structure directly enhanced the mechanical integrity of the polymer network. UV treatment also increased α-helix content (35.36%), albeit less pronounced than ultrasound. In contrast, microwave treatment induced only minor variations relative to the control. These findings suggest that physical-field treatments, particularly ultrasound, promote protein conformational rearrangements that contribute to improved mechanical and barrier properties [3].

**Table 1:** Quantitative analysis of relative contents of protein secondary structures under different physical treatments.

**Sample**	**α-helix**	**β-sheet**	**β-turn**	**Random coil**
**Control**	31.82%	37.29%	13.29%	17.60%
**US**	36.68%	31.79%	13.95%	17.58%
**UV**	35.36%	34.72%	13.17%	16.75%
**MW**	32.56%	36.63%	13.04%	17.77%

The deconvoluted amide I region spectra (Fig. 3C) were analyzed following well-established protocols for protein secondary structure determination [5], [17]. Prior to curve fitting, second-derivative spectra and Fourier self-deconvolution were applied to identify the number and initial positions of the underlying components. The amide I band (1600–1700 cm^−1^) was then fitted with multiple Gaussian peaks, with the full width at half maximum (FWHM) constrained to 8–15 cm^−1^ to avoid over-fitting. The goodness of fit was evaluated by minimizing the residual standard error. The characteristic sub-bands were assigned as follows: β-sheet structures (∼1612, 1624, 1675, and 1685 cm^−1^), random coil (∼1634 cm^−1^), α-helix (∼1645 and 1656 cm^−1^), and β-turn (∼1665 cm^−1^), based on literature reports ((Byler et al., 1986; Jilie et al., 2007). Compared with the control, both ultrasound and UV treatments increased the relative area of the α-helix-associated bands (∼1645–1656 cm^−1^), accompanied by a reduction in β-sheet content. In contrast, microwave-treated films exhibited spectral characteristics similar to those of the control. Overall, these spectral changes confirm that ultrasound and UV treatments enhance molecular ordering within the protein matrix [33].

### 3.4 X–ray photoelectron spectroscopy (XPS) analysis

X-ray photoelectron spectroscopy (XPS) was employed to investigate the surface chemical composition and bonding configurations of CP–gelatin films. The survey spectra (Fig. 3D) confirmed the presence of carbon (C 1 s, ∼285 eV), nitrogen (N 1 s, ∼400 eV), and oxygen (O 1 s, ∼532 eV), originating primarily from gelatin amino acids and the polysaccharide backbone of cactus CP. Following ultrasound treatment, the surface nitrogen content increased to 11.26%, while oxygen content decreased to 17.17%, indicating that acoustic cavitation promoted the exposure of gelatin-rich domains at the film surface. In contrast, UV and microwave treatments increased oxygen content (32.41% and 35.89%, respectively) and reduced nitrogen content (7.62% and 6.88%, respectively), suggesting preferential surface enrichment of polysaccharide components or reduced exposure of protein domains [54]. These results are consistent with FTIR analysis, confirming that each physical field induces distinct surface chemical rearrangements.

Deconvolution of the high-resolution C 1 s spectra (Fig. 3E) further clarified interfacial chemical modifications. The US-treated film exhibited an increased C–O/C–N component (∼286.2 eV), indicating enhanced intermolecular interactions and improved dispersion of CP within the gelatin matrix. In contrast, the UV-treated film showed an increased C=O/N–C=O contribution (∼288.0 eV), consistent with photo-oxidation and crosslinking of protein side chains. Microwave treatment induced slight binding-energy shifts, likely arising from localized dehydration and rearrangement of polar groups under rapid volumetric heating [6]. These chemical changes directly contributed to the observed variations in wettability and barrier performance.

### 3.5 X-ray diffraction analysis

The crystalline structure of CP–gelatin films was analyzed by XRD (Fig. 4A). All samples exhibited a broad diffraction halo at 2θ = 20°–23°, indicating a predominantly amorphous structure with limited short-range ordering [26]. However, physical treatments significantly modified peak position and intensity (Fig. 4B).

**Fig. 4:**
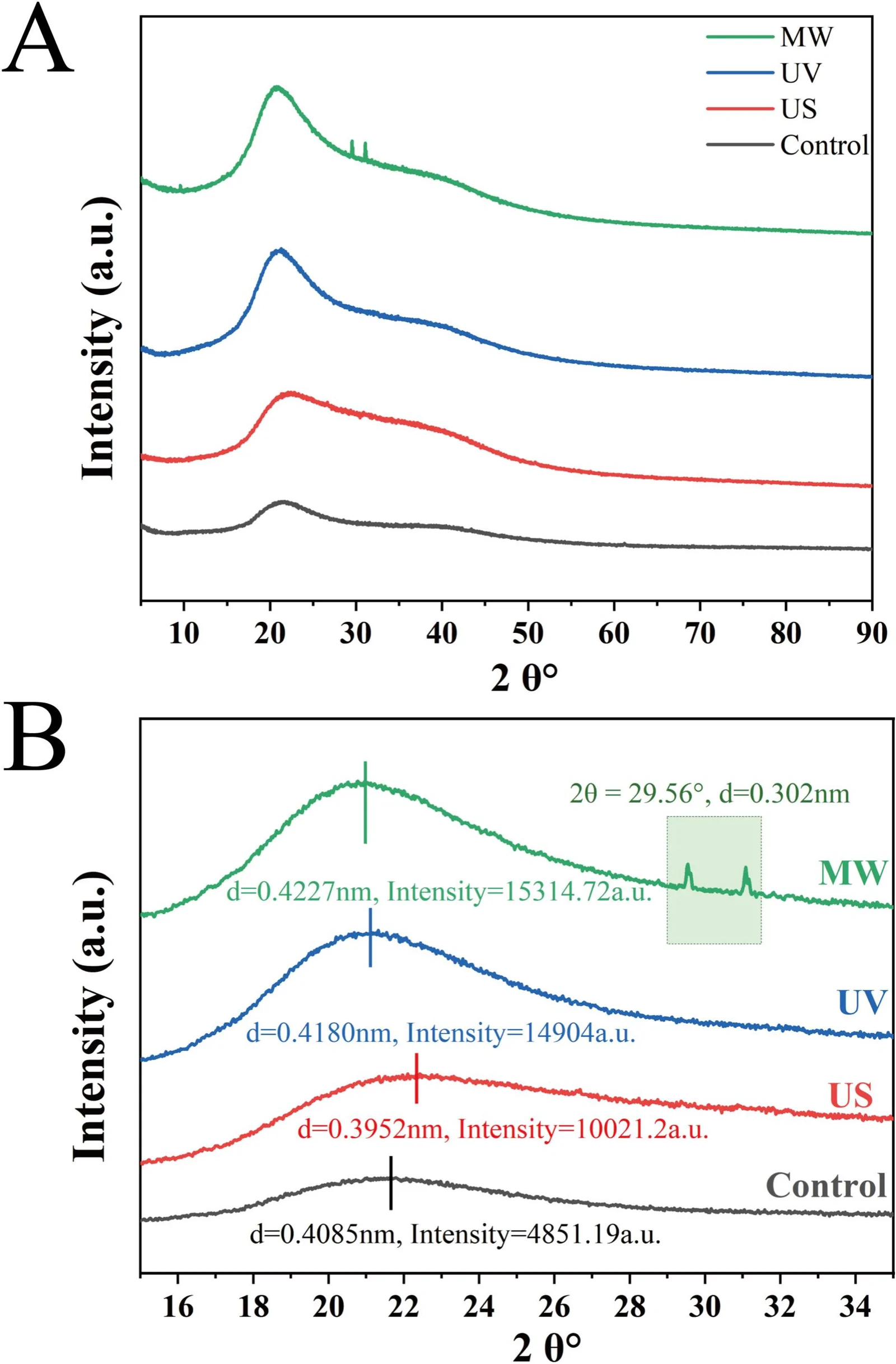
(A) X–ray diffraction (XRD) patterns of cactus polysaccharide–gelatin composite films under different physical treatments. (B) Enlarged view of the XRD patterns in the selected 2θregion (15 – 30°), highlighting the peak shifts and the appearance of a sharp crystalline reflection.

For the ultrasound-treated film, the diffraction peak shifted to a higher angle (2θ = 22.48°) with reduced d-spacing (0.3952 nm), indicating tighter molecular packing and stronger intermolecular interactions. According to Bragg's law (nλ = 2d sin θ), this higher-angle shift corresponds to decreased interplanar spacing, reflecting denser chain packing, which agrees with the compact morphology observed in SEM. In contrast, UV treatment shifted the peak to a lower angle (2θ = 21.24°, d = 0.4180 nm), suggesting increased interchain spacing due to crosslinked network formation that locks chains in a more open conformation. The microwave-treated film exhibited a distinct reflection at 2θ = 29.56° (d = 0.302 nm), indicating localized ordered domains formed by rapid volumetric heating and dehydration-induced molecular rearrangement [49], [53]. To quantitatively compare crystalline ordering, the relative crystallinity index (CrI) was calculated using the peak height method. The CrI values for control, US, UV, and MW were 34.3%, 46.3%, 39.0%, and 42.2%, respectively. US treatment resulted in the highest CrI (46.3%), consistent with reduced d-spacing and tighter packing. MW also showed a notable increase (42.2%) due to localized crystallization, while UV exhibited the smallest increase (39.0%), consistent with surface-limited crosslinking. These structural modifications directly influenced macroscopic properties: denser packing in US-treated films contributed to enhanced tensile strength (20.53 MPa), reduced WVP (1.81 × 10^−7^), and lower swelling (99.0%), while increased interchain spacing in UV-treated films weakened mechanical performance. Overall, physical treatments modulated intermolecular packing and crystalline ordering without disrupting the predominantly amorphous matrix, and the type and extent of structural reorganization critically determined the final performance of the composite films [52].

### 3.6 Thermogravimetric analysis and differential scanning calorimetry analysis

TGA was used to evaluate the thermal stability of CP–gelatin films. All samples exhibited three degradation stages: moisture evaporation (30–120 °C), main polymer decomposition (120–400 °C), and char formation (>400 °C) (Fig. 5A). The control film showed a Tmax of 234.9 °C with 81.04% total weight loss. After ultrasound treatment, Tmax decreased to 224.1 °C, with reduced weight loss (80.81%) and increased residual mass (19.19%). UV treatment maintained similar Tmax (234.7 °C) but showed the highest weight loss (81.98%), while MW treatment exhibited slightly higher Tmax (236.0 °C) with 83.26% weight loss and 16.74% residue.

**Fig. 5:**
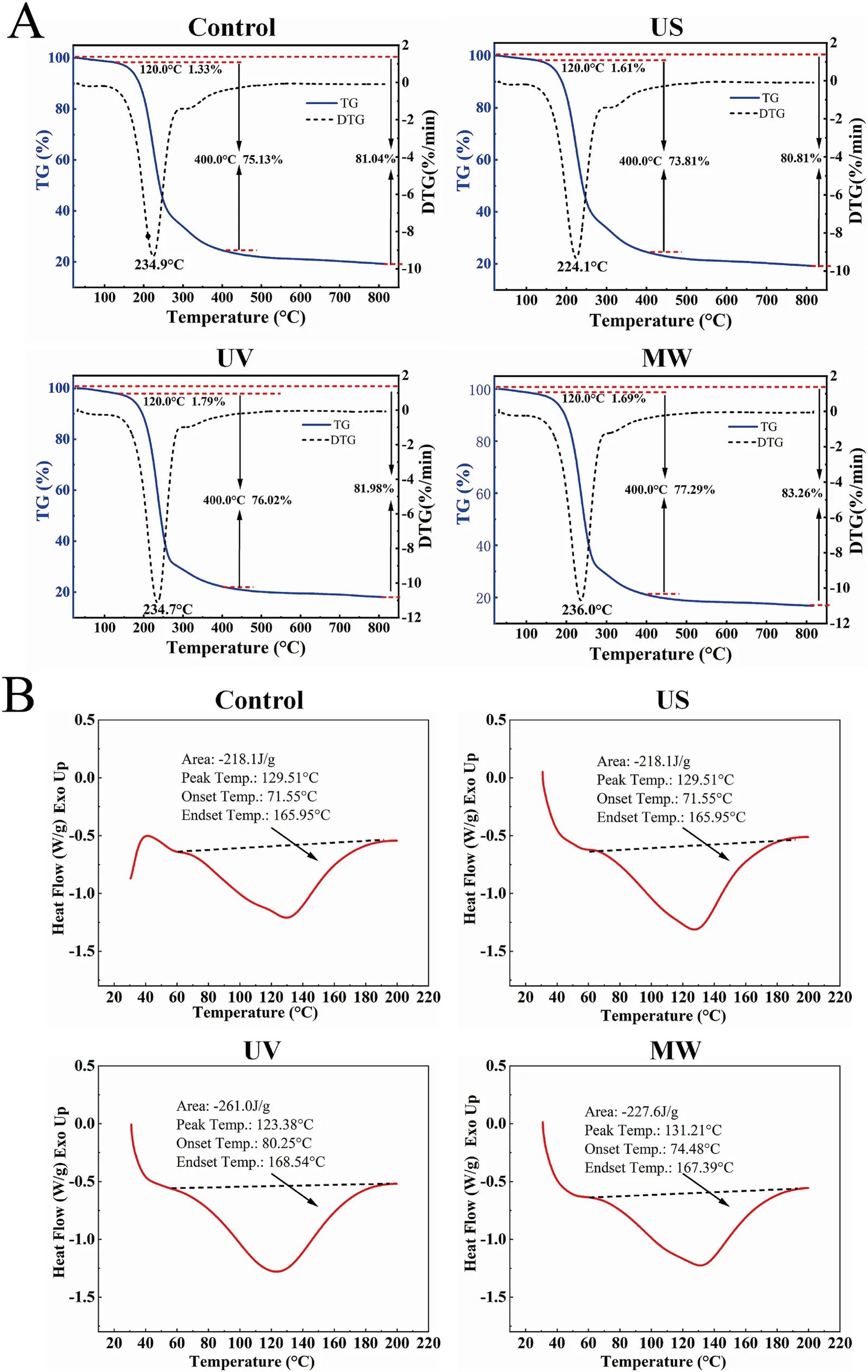
(A) Thermogravimetric (TGA) curves of cactus polysaccharide–gelatin composite films under different physical treatments. (B) Differential scanning calorimetry (DSC) thermograms of cactus polysaccharide–gelatin composite films under different physical treatments.

The reduction in Tmax for US-treated films suggests partial chain scission induced by acoustic cavitation, resulting in slightly lower thermal resistance. However, the higher residual mass indicates enhanced char formation due to stronger intermolecular interactions [36]. Overall, the TGA results suggest that ultrasound treatment slightly reduced the decomposition temperature while increasing char yield, whereas UV and MW treatments promoted greater mass loss during thermal degradation.

The thermal behaviour of CP-gelatin composite films subjected to different physical treatments was further investigated by differential scanning calorimetry (DSC). All samples exhibited a single endothermic transition within the temperature range of 120–140 °C (Fig. 5B), which can be attributed to the helix-to-coil transition of gelatin together with thermal disruption of the polysaccharide–protein network. The control film exhibited an endothermic enthalpy (ΔH) of 218.1 J/g and a peak temperature (Tpeak) of 129.5 °C. The UV-treated sample showed the highest enthalpy (261.0 J/g) and the lowest Tpeak (123.4 °C), whereas the US-treated film exhibited a lower onset temperature (Tonset = 73.4 °C) and an enthalpy of 249.4 J/g. The MW-treated film displayed intermediate thermal behaviour, with an elevated Tonset (74.5 °C) and a Tpeak of 131.2 °C.

The increased endothermic enthalpy observed in the UV- and MW-treated films suggests that these treatments promoted stronger intermolecular interactions within the gelatin–polysaccharide matrix, thereby requiring greater energy for structural disruption. However, the lower Tpeak observed for the UV-treated film indicates that photo-induced crosslinking may have altered the cooperative stability of the gelatin triple-helix structure. The intermediate thermal parameters observed for the MW-treated film suggest a moderate influence of microwave-induced thermal effects. In contrast, the US-treated film exhibited a lower Tonset together with reduced thermal stability parameters, indicating that ultrasonic cavitation may have partially disrupted the ordered structure of the polymer network [43]. Nevertheless, the relatively high enthalpy value (249.4 J/g) indicates that a considerable degree of structural organization was retained after ultrasound treatment.

These DSC results align with the TGA findings, showing distinct thermal responses to different physical fields. The slight reduction in Tmax for US-treated films is consistent with reports that excessive ultrasonic treatment can degrade properties [4], and ultrasonic cavitation promotes fracture of chemical chain segments, which may weaken the polymer network and reduce thermal resistance [55]. Notably, UV-exposed gelatin films have been reported to maintain unchanged thermal stability [22], which supports our observation that UV treatment kept Tmax comparable to control. In contrast, while microwave extraction can influence gelatin stability, the slight Tmax increase observed in our MW group suggests structural reorganization and remains a distinctive finding of this work.

### 3.7 Thickness and mechanical properties analysis

The thickness of CP–gelatin composite films was measured using a digital micrometer (Fig. 6A). The control film exhibited an average thickness of 0.400 ± 0.012 mm. Following ultrasound treatment, thickness decreased to 0.363 ± 0.010 mm, which can be attributed to the cavitation-induced compaction effect that reduces microvoids and promotes the formation of a denser polymer network. Microwave treatment also reduced film thickness (0.373 ± 0.007 mm), whereas UV treatment resulted in a more moderate decrease (0.387 ± 0.008 mm). The thickness of the films followed the order: Control (0.400 ± 0.012 mm) > UV (0.387 ± 0.008 mm) > MW (0.373 ± 0.007 mm) > US (0.363 ± 0.010 mm). All treated films exhibited significantly lower thickness than the control (p < 0.05), while the difference between UV and MW, and between MW and US, was not statistically significant (p > 0.05). These results indicate that physical treatments, enhanced matrix compactness.

**Fig. 6:**
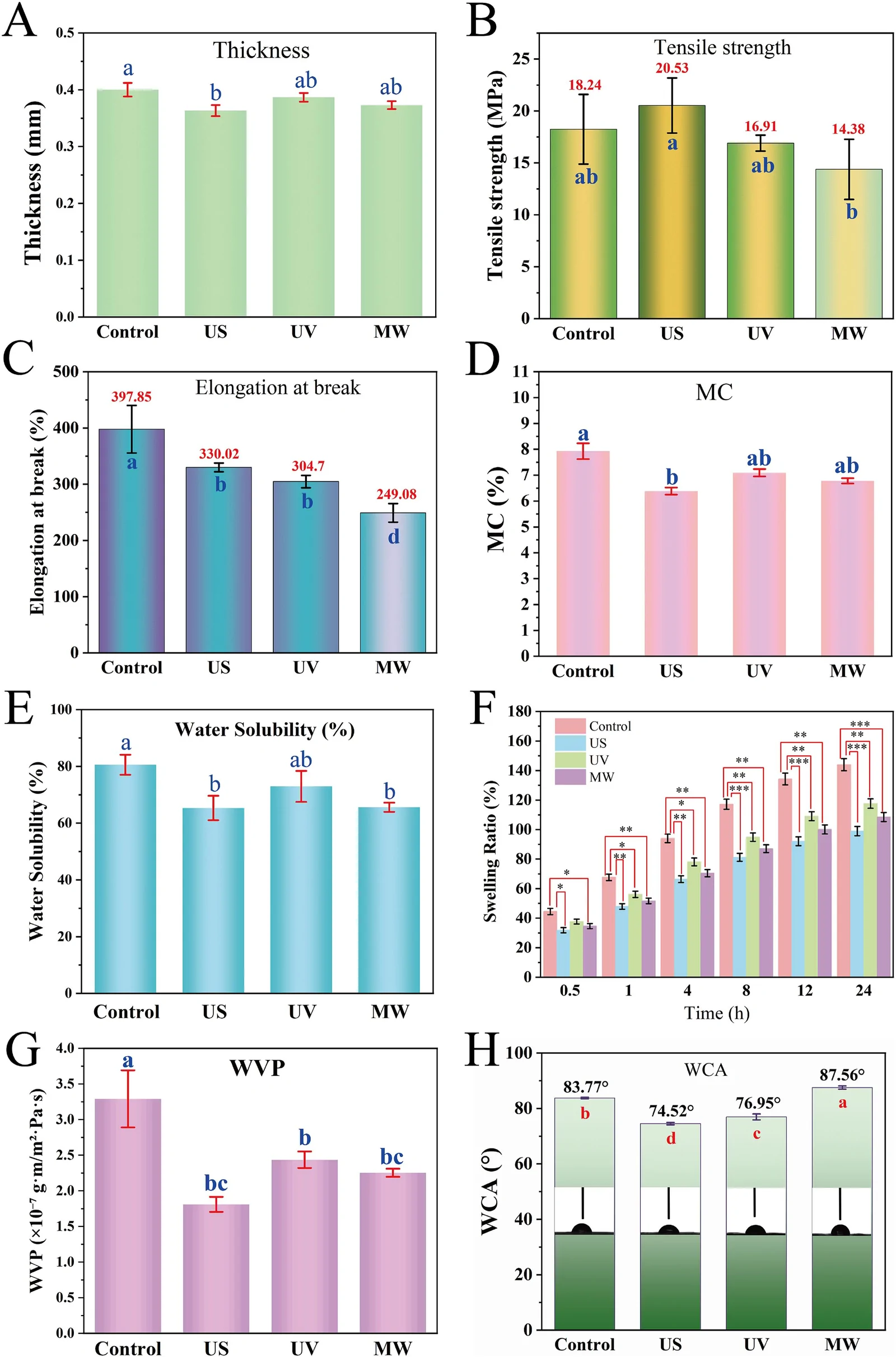
Physicochemical and mechanical properties of cactus polysaccharide–gelatin composite films under different physical treatments. (A) Film thickness, (B) Tensile strength (TS), (C) Elongation at break (EB), (D) Moisture content (MC), (E) Water solubility (WS), (F) Swelling ratio, (G) Water vapour permeability (WVP), (H) Water contact angle. All data are presented as mean ± standard deviation (n = 3). Different letters indicate significant differences among treatments (p < 0.05).

The tensile strength (TS) and elongation at break (EB) of the control film were 18.24 MPa and 397.85%, respectively (Fig. 6A and 6B). Ultrasound treatment increased TS to 20.53 MPa while reducing EB to 330.02%, which can be attributed to cavitation-induced densification and enhanced hydrogen bonding, consistent with [55]. Remarkably, the tensile strength of our ultrasound-treated film (20.53 MPa) is competitive with or even superior to that of gelatin-based films reinforced with zero-dimensional nanomaterials, such as carbon dots [28]. This suggests that ultrasound-induced physical restructuring—through densification and enhanced intermolecular bonding—can effectively activate the intrinsic reinforcement potential of the polymer matrix itself, offering a cleaner and potentially more scalable alternative to nanomaterial-based modification strategies. Despite the improvement, the tensile strength (20.53 MPa) remains moderate compared to synthetic or chemically crosslinked films (>30 MPa), representing a limitation for high-strength applications, though acceptable for food packaging.

In contrast, [11] reported that UV-treated gelatin films showed a decrease in tensile strength from approximately 25 MPa to 18 MPa, accompanied by a significant reduction in elongation, which is consistent with our UV results. Similarly, [40] observed that microwave treatment reduced the mechanical integrity of polymer films due to localized thermal effects, which aligns with our MW results (14.38 MPa and 249.08%). These findings demonstrate that ultrasound treatment under the applied conditions improved film strength, whereas UV and MW treatments reduced both strength and flexibility, highlighting the importance of optimizing treatment conditions for different physical fields.

### 3.8 Moisture content and water solubility analysis

The moisture content (MC) of CP–gelatin films decreased after all physical treatments (Fig. 6D). The control film exhibited the highest MC (7.93 ± 0.61%), followed by UV (7.09 ± 0.28%), MW (6.78 ± 0.17%), and US (6.38 ± 0.23%) treatments. Significant differences were observed between the control and US-treated films (p < 0.05), while the MC values of UV- and MW-treated films were not significantly different from either the control or the US-treated film (p > 0.05). The most pronounced reduction in the US-treated film can be attributed to cavitation-induced network densification, which limits water uptake and enhances matrix compactness [50]. Similar trends have been reported for ultrasound-treated protein-based films, where reductions in MC and WVP are accompanied by improved mechanical strength [19]. Ultrasonic treatment of gelatin emulsions has also been shown to reduce thickness, WVP, and water solubility, thereby improving overall film performance under optimized conditions (L. [55]. For microwave treatment, although direct reports on gelatin systems remain limited, the observed reduction in MC is consistent with microwave-induced enhancement of polymer crosslinking and barrier properties [7]. UV treatment produced a more moderate reduction in MC, consistent with its predominantly surface-limited effects, as previously reported where UV irradiation reduces hydrophilicity and water solubility to a lesser extent [42]. Reduced moisture content enhances the dimensional stability of edible films during storage [50].

As shown in Fig. 6E, the decrease in water solubility (WS) of CP–gelatin films is consistent with reports on physically modified biopolymer systems. The strongest reduction in the US-treated film reflects enhanced intermolecular interactions and improved network cohesion, in agreement with previous studies showing that ultrasonication significantly decreases WS while improving mechanical performance [55]. UV-treated gelatin-based films have also been reported to exhibit reduced water solubility due to photo-induced crosslinking. For microwave treatment, although studies in gelatin systems remain limited, the observed reduction in WS is consistent with non-thermal enhancement of polymer crosslinking and barrier properties [8]. Overall, physical treatments, particularly ultrasound, effectively reduced water solubility and improved resistance to aqueous environments, which is beneficial for food-packaging applications under high humidity conditions.

### 3.9 Swelling index, water vapor permeability and water contact angle analysis

The swelling behaviour of CP–gelatin composite films subjected to different physical treatments was evaluated by measuring weight gain (%) during immersion in distilled water for up to 24 h (Fig. 6F). All films exhibited a rapid increase in swelling during the first 8 h, followed by a gradual plateau. The control film showed the highest swelling throughout the testing period, reaching 144.0 ± 4.1% after 24 h, indicating a loose and highly water-permeable network structure. In contrast, all treated films exhibited significantly lower swelling indices (p < 0.05). The US-treated film showed the lowest swelling (99.0 ± 3.2% at 24 h), which can be attributed to cavitation-induced network densification that restricts water diffusion. MW treatment also reduced swelling (108.5 ± 3.1%), albeit less effectively than ultrasound, while UV treatment showed intermediate values (117.7 ± 3.2%). These results are consistent with the structural changes observed by XPS and XRD, confirming that physical treatments, particularly ultrasound, enhance network integrity and water resistance. The water vapour permeability (WVP) followed the order: Control (3.29 × 10^−7^ g·m^−2^·Pa^−1^·s^−1^) > UV (2.44 × 10^−7^) > MW (2.25 × 10^−7^) > US (1.81 × 10^−7^) (Fig. 6G). Significant differences (p < 0.05) were observed between the control and all treated films, as well as between US and UV treatments. However, the difference between MW and UV was not statistically significant (p > 0.05). All treated films exhibited significantly reduced WVP, indicating improved resistance to water vapour transmission. The lowest WVP observed in the US-treated film is attributed to enhanced matrix homogeneity and compact network formation induced by ultrasonic cavitation (L. [55]. MW treatment also reduced WVP, likely due to thermally induced polymer-chain rearrangement and strengthened intermolecular interactions between gelatin and cactus polysaccharides. In contrast, UV treatment produced a weaker reduction, suggesting that crosslinking effects were mainly confined to the film surface [22]. The reduced WVP values are consistent with lower moisture content and water solubility, confirming that physical treatments, particularly ultrasound, effectively improve moisture barrier performance in CP–gelatin films.

The decrease in WCA after US treatment (from 83.77° to 74.52°) can be directly correlated with the XPS-observed surface nitrogen enrichment (from 7.62% to 11.26%). As nitrogen is predominantly derived from the gelatin component (rich in polar amino acid residues such as serine, threonine, and glutamine), its increased surface exposure indicates that acoustic cavitation disrupted the hydrophobic gelatin-polysaccharide aggregation, exposing additional polar functional groups (hydroxyl, carboxyl, and amino moieties) at the film surface [47]. This surface chemical rearrangement, rather than a change in overall hydrophilicity of the bulk material, accounts for the increased wettability observed in US-treated films. In contrast, MW treatment increased surface oxygen content (from 28.65% to 35.89%) while decreasing nitrogen content (from 7.62% to 6.88%), suggesting that thermal-induced rearrangement preferentially oriented hydrophobic gelatin domains toward the air interface, thereby increasing WCA (87.56°).

### 3.10 Optical properties analysis

The optical properties of CP–gelatin films were significantly influenced by different physical treatments (Table 2). The US-treated film exhibited the highest lightness (L* = 78.1) and the lowest b* value (10.0), indicating a brighter appearance with reduced yellow coloration. In contrast, the UV-treated film showed a lower lightness value (L* = 63.7), resulting in a darker appearance. The total color difference (ΔE) was highest for the MW-treated film (5.6) and lowest for the UV-treated film (1.1), reflecting treatment-dependent variations in optical appearance.

**Table 2:** Optical properties of cactus polysaccharide–gelatin composite films under different physical treatments (Control, US, UV, MW).

	L	a	b	ΔE	T_280_	T_660_
Control	68.4 ± 1.4^b^	−1.4 ± 0.0^a^	−17.5 ± 0.8^a^	3.1 ± 0.5^b^	0.0 ± 0.0^a^	71.3 ± 1.6^b^
US	78.1 ± 1.8^c^	−0.7 ± 0.0^b^	−10.0 ± 1.0^c^	2.1 ± 0.7^ab^	0.0 ± 0.0^a^	53.0 ± 3.4^a^
UV	63.7 ± 1.7^a^	−0.8 ± 0.3^b^	−15.1 ± 0.6^b^	1.1 ± 0.8^a^	0.2 ± 0.2^a^	74.5 ± 1.2^b^
MW	66.6 ± 3.9^ab^	−0.9 ± 0.0^b^	−14.5 ± 0.5^b^	5.6 ± 1.1^c^	0.1 ± 0.1^a^	71.1 ± 4.9^b^

Data are expressed as mean ± SD (n = 3). Different superscript letters in the same column indicate significant differences (p < 0.05). L: lightness; a: redness/greenness; b*: yellowness/blueness; ΔE: total color difference; T ∼ 280 ∼ and T ∼ 660∼: transmittance at 280 nm and 660 nm, respectively.

All films exhibited excellent UV-blocking ability, with transmittance at 280 nm approaching zero, indicating that physical treatments did not compromise ultraviolet protection. At 660 nm, visible-light transmittance varied significantly: the US-treated film showed the lowest value (53.0%), whereas the UV-treated film exhibited the highest transparency (74.5%). These results indicate that physical treatments enable tunable optical properties, which may be advantageous for different packaging applications requiring controlled light transmission.

The UV–Vis transmission spectra of CP–gelatin films (Fig. 7A and Fig. 7B) confirmed strong UV-blocking performance across all samples. The superior UV-shielding performance of the US-treated films, particularly in the UV-B region, is consistent with previous reports showing that ultrasonication enhances UV-barrier properties of biopolymer films while maintaining acceptable transparency (X. H. [58]. This improvement can be attributed to ultrasound-induced structural densification, which reduces light scattering and enhances UV absorption. In contrast, UV treatment reduced UV-blocking efficiency, likely due to photo-induced degradation of UV-absorbing aromatic amino acids such as tyrosine and tryptophan in gelatin [45]. The distinct transmittance profiles observed among treatments reflect different modification mechanisms, highlighting the importance of process control in tailoring optical functionality.

**Fig. 7:**
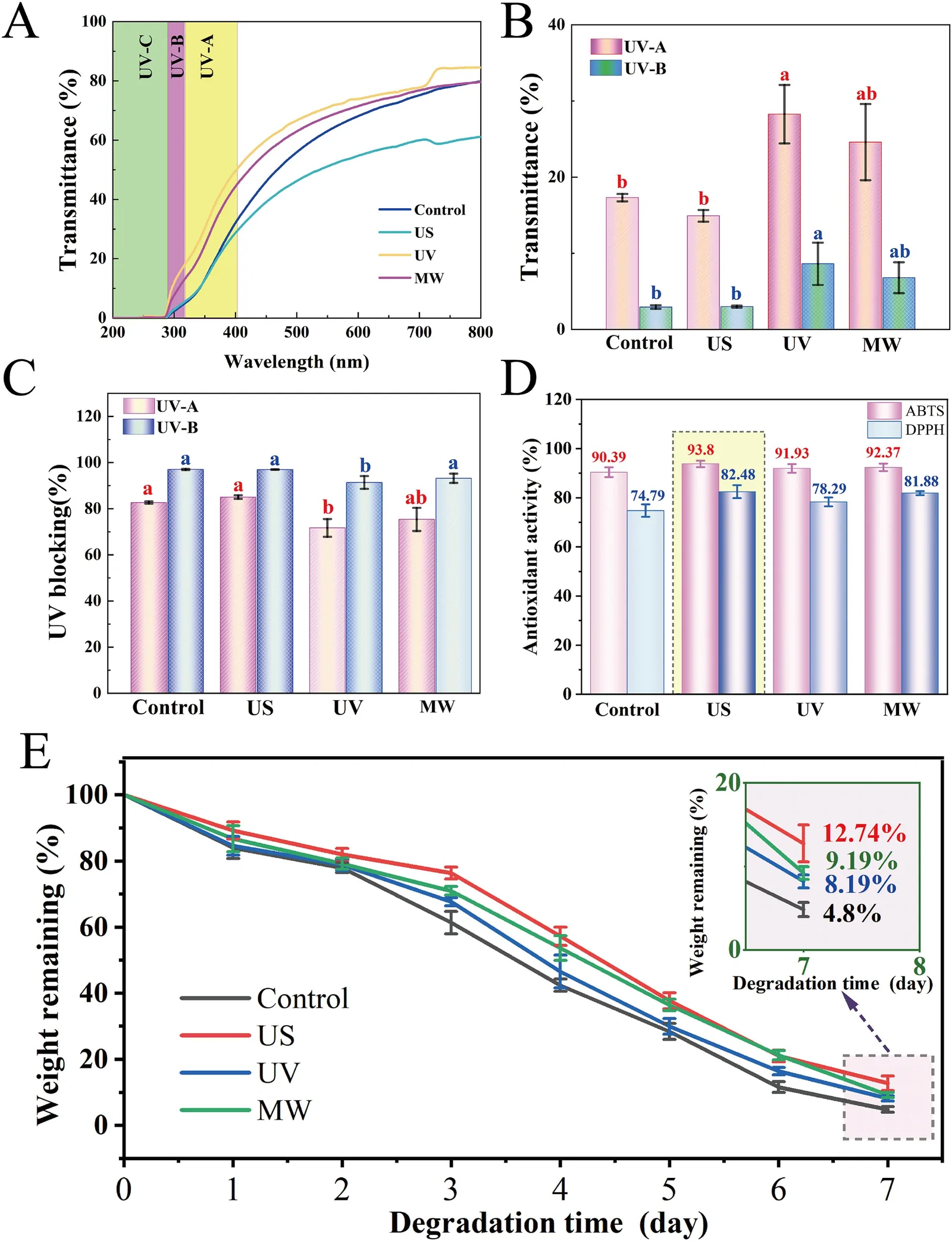
(A) UV–visible transmittance spectra of cactus polysaccharide-gelatin composite films under different physical treatments. (B) UV-A (315–400 nm) and UV-B (280–315 nm) transmittance of the films. (C) UV-blocking properties (UV-A and UV-B blocking efficiencies) of the films. (D) Antioxidant activities (ABTS and DPPH radical scavenging activities) of the films. (E) Weight remaining of the films during soil biodegradation. All data are presented as mean ± standard deviation (SD) from three independent measurements (n = 3). Where error bars are not visible, they are smaller than the symbol size. Different letters indicate significant differences among treatments (p < 0.05).

Fig. 7C summarizes UV-A and UV-B blocking efficiencies of CP–gelatin films. The US-treated film exhibited the highest UV-A blocking efficiency (85.07%), followed by the Control (82.68%), MW (75.40%), and UV (71.72%) films. For UV-B radiation, all films demonstrated high blocking efficiencies exceeding 91%, with the Control (97.04%) and US-treated (96.99%) films showing the highest values, while the UV-treated film showed the lowest value (91.37%). These results indicate that ultrasound treatment effectively maintained UV-B shielding while enhancing UV-A protection compared with the control.

In contrast, UV treatment reduced UV-blocking efficiency in both UV-A and UV-B regions, likely due to photo-induced modification of UV-absorbing components within the film matrix [22]. Overall, the US-treated film exhibited the most comprehensive UV-shielding performance, highlighting its potential for active food packaging applications requiring efficient ultraviolet protection.

### 3.11 Antioxidant activity analysis

The antioxidant activities of CP–gelatin composite films subjected to different physical treatments (Control, US, UV, and MW) were evaluated using ABTS and DPPH radical-scavenging assays. As shown in Fig. 7D, the control film exhibited ABTS and DPPH scavenging activities of 90.39% and 74.79%, respectively. All physical treatments enhanced radical-scavenging activity compared with the control. The US-treated film exhibited the highest antioxidant activity, with ABTS and DPPH scavenging efficiencies of 93.80% and 82.48%, respectively, which were significantly higher than those of the control (90.39% and 74.79%) and UV-treated films (91.93% and 78.29%) (p < 0.05). MW treatment also significantly improved antioxidant performance (ABTS: 92.37%; DPPH: 81.88%) compared with the control, though its DPPH scavenging activity was slightly but not significantly lower than that of the US-treated film (p > 0.05). The enhanced antioxidant activity may be attributed to increased accessibility or release of phenolic compounds and other antioxidant constituents from the cactus polysaccharide matrix induced by physical treatments [9]. Ultrasound-induced cavitation disrupts molecular aggregates and promotes redistribution of antioxidant components within the gelatin network. In addition, structural rearrangement induced by ultrasound may expose more functional groups capable of hydrogen or electron donation, thereby enhancing radical-scavenging efficiency. Although microwave treatment also improved antioxidant activity, localized thermal effects may have partially affected heat-sensitive antioxidant compounds, resulting in a slightly lower enhancement than ultrasound. In contrast, UV treatment showed the weakest improvement, likely due to its predominantly surface-limited modification effects. Overall, physical treatments, particularly ultrasound, not only improved structural and barrier properties but also significantly enhanced antioxidant functionality. These combined improvements highlight the potential of US-treated CP–gelatin films for active food packaging applications requiring both protective and antioxidant functions.

### 3.12 Biodegradability analysis

The soil biodegradation behavior of CP–gelatin films subjected to different physical treatments was evaluated by monitoring residual mass over 7 days (Fig. 7E). All films exhibited continuous mass loss during soil burial, confirming their biodegradable nature. However, degradation rates varied significantly depending on treatment. The control film degraded most rapidly, retaining only 4.80 ± 0.84% of its initial mass after 7 days. In contrast, the US-treated film showed the slowest degradation, with 12.74 ± 2.10% residual mass remaining, followed by MW-treated (9.19 ± 0.76%) and UV-treated films (8.19 ± 0.78%).

The improved resistance to biodegradation in the US-treated film may be attributed to the formation of a denser and more compact polymer network, which limits water penetration and microbial accessibility. MW treatment also enhanced degradation resistance, likely due to thermally induced molecular rearrangement and strengthened intermolecular interactions [44]. UV treatment produced a more moderate effect, consistent with its primarily surface-oriented modification mechanism. The biodegradation resistance followed the order: US > MW > UV > Control, which is consistent with trends observed for water vapour permeability, swelling index, and water solubility. These results indicate that physical treatments simultaneously influence moisture resistance and biodegradation behavior. Overall, ultrasound treatment effectively extends film durability while maintaining biodegradability, making it suitable for applications requiring controlled environmental degradation.

### 3.13 Mechanistic summary of structure–property relationships

Based on the multiscale characterization results, the distinct effects of the three physical-field treatments on CP-gelatin films can be rationalized through a unified mechanistic framework:

Ultrasound acts primarily through cavitation-induced mechanical forces, which disrupt molecular aggregates, promote chain rearrangement, and enhance intermolecular hydrogen bonding. This leads to a denser, more ordered structure with reduced interchain spacing and increased α-helix content, ultimately improving mechanical strength and barrier properties while maintaining adequate flexibility.

UV irradiation operates via photochemical crosslinking, primarily at the film surface, which restricts chain mobility and increases interchain spacing in a more open crosslinked network. While this improves surface stability and optical transparency, it compromises mechanical integrity and does not substantially enhance bulk barrier properties.

Microwave treatment exerts thermal effects through rapid volumetric heating, inducing localized chain mobility and dehydration. This promotes partial crystallization and surface hydrophobic domain orientation, enhancing surface hydrophobicity, but the non-uniform heating also introduces nanoscale heterogeneity and thermal degradation that reduce overall mechanical performance.

This mechanistic framework provides a rational basis for selecting physical-field treatments based on the specific functional requirements of target packaging applications.

## Conclusions

This study successfully fabricated CP–gelatin composite films and systematically evaluated the effects of ultrasound (US), ultraviolet (UV), and microwave (MW) treatments on their structural, physicochemical, mechanical, thermal, and functional properties. The results demonstrated that each physical field induced distinct modifications in film structure and performance. Among the investigated treatments, ultrasound exhibited the most pronounced overall enhancement. US treatment promoted a homogeneous and compact microstructure, strengthened intermolecular interactions, increased α-helix content, and induced surface nitrogen enrichment, resulting in improved molecular packing. These structural changes translated into enhanced tensile strength and significantly improved barrier properties, including reduced moisture content, water solubility, swelling index, and water vapour permeability, along with superior antioxidant activity and UV-shielding performance. In comparison, UV treatment mainly induced surface crosslinking and improved optical transparency, while MW treatment promoted localized crystalline formation and increased surface hydrophobicity. Under the specific processing parameters employed, ultrasound exhibited the most pronounced overall enhancement among the three treatments. However, rather than claiming absolute superiority of one technology over others, the findings are confined to the specific processing parameters employed, and comprehensive parametric optimization of each technology was beyond the scope of this investigation. Nevertheless, the results highlight the potential of US-treated films as sustainable active packaging materials and provide valuable insights into the application of physical-field technologies for designing advanced biodegradable hydrocolloid systems. Future studies should focus on optimizing processing parameters for each technology and evaluating film performance under real-world food packaging conditions.

## CRediT authorship contribution statement

**Shikuan Zhao:** Writing – review & editing, Writing – original draft. **Muhammad Aaqil:** Writing – review & editing. **Feng Zhang:** Validation. **Rui Zhou:** Validation. **Siyu Zhou:** Visualization. **Yuanfeng Chen:** Data curation. **Cheng Gong:** Validation. **Huiqing Luo:** Supervision. **Chongye Fang:** Formal analysis. **Cunchao Zhao:** Funding acquisition.

## Funding

This study was supported by Special Project on Building a Science and Technology Innovation Center for South and Southeast Asia-International Joint Innovation Platform of Yunnan Province (202503AP140010).

## Declaration of competing interest

The authors declare that they have no known competing financial interests or personal relationships that could have appeared to influence the work reported in this paper.

## Appendix A Supplementary data

Supplementary Data 1 This document contains the detailed experimental methods (Sections 1–6) and Figure S1, which presents representative force-displacement curves of the CP-gelatin composite films under different physical treatments.
