# Supplementary material for: Comparative effects of ultrasound, ultraviolet, and microwave treatments on the structure-property relationships of cactus polysaccharide-gelatin composite films
Source: Ultrason Sonochem. 2026 Sep 23;133:108079. doi: 10.1016/j.ultsonch.2026.108079 (PMC13635943; doi:10.1016/j.ultsonch.2026.108079)
Supplement: Supplementary file 1 [file mmc1.docx]

**Comparative Effects of Ultrasound, Ultraviolet, and Microwave Treatments on the Structure–Property Relationships of Cactus Polysaccharide–Gelatin Composite Films**

Shikuan Zhao^a,1^, Muhammad Aaqil^a,1^, Feng Zhang^a^, Rui Zhou^a^, Siyu Zhou^a^, Yuanfeng Chen^a^, Huiqing Luo^a^, Cheng Gong^a*^, Chongye Fang^a*^, Cunchao Zhao^a,b,c*^

a: College of Food Science and Technology, Yunnan Agricultural University, Kunming, 650201, China.

b: Yunnan Plateau Characteristic Agricultural Industry Research Institute, Yunnan Agricultural University, Kunming, 650201, China.

c: Yunnan International Joint Laboratory of China-Cambodia Signiture Agro-Products Green Development, Kunming, 650201, China.

The first author^1^, Mr Shikuan Zhao (E-mail: 15153216972@163.com) and Mr Muhammad Aaqil (aaqilkhan26@gmail.com).

^1^ These authors contributed equally to this work.

The corresponding author*, Mr CunChao Zhao (E-mail: zcc272744372@163.com).

1. **Preparation and Compositional Analysis of cactus polysaccharide (CP)**

Fresh cactus specimens were rinsed, lyophilized, pulverized, and then sieved using a 60‑mesh screen. The resulting powder (500 g) was defatted with petroleum ether at 25 °C for 4 h. Subsequently, 100 g of the defatted powder was extracted with 5 L of distilled water at 100 °C for 4 h. Following centrifugation at 8000 × g for 20 min, the resulting supernatant was harvested, reduced to 10% of its initial volume via rotary evaporation, and then mixed with ethanol to a final concentration of 80% (v/v) and kept at 4 °C for 12 h to allow precipitation. The precipitate was recovered by centrifugation, deproteinized four times using the Sevag method, dialyzed, and freeze-dried to obtain cactus polysaccharide (CP) (Li et al., 2025). The following equation was used to determine the total polysaccharide content:

Total polysaccharide content (%) =$\text{ }\frac{\text{Mass of total polysaccharides in the CP extract (g)}}{\text{Mass of the CP extract (g)}}$ × 100%

Protein content was determined using the Coomassie Brilliant Blue method with bovine serum albumin (BSA) as the standard (Fangfang et al., 2022). Standard BSA solutions (0–100 μg/mL) were prepared to establish a calibration curve. Briefly, 5 mL of Coomassie Brilliant Blue reagent was added to 1 mL of each standard or sample solution. The mixture was incubated for 5 min at ambient temperature, after which the absorbance was read at 595 nm. The protein content of the sample (2.5 mg/mL) was then determined using the calibration curve.

The total phenolic content was quantified using the Folin–Ciocalteu assay, with gallic acid serving as the reference standard (Pérez et al., 2023). In brief, 100 μL of each standard or sample solution was combined with 400 μL of distilled water and 100 μL of Folin–Ciocalteu reagent. After a 6‑min reaction, 1.0 mL of 7% Na₂CO₃ and 800 μL of distilled water were added, and the mixture was incubated for 90 min. The absorbance at 760 nm was then recorded. The sample solution (2.5 mg/mL) was processed under the same conditions, and the total phenolic content was derived from the standard calibration curve.

2. **Fabrication of multifunctional CP‑gelatin films subjected to different physical treatments**

A solution‑casting technique was adopted to fabricate the CP‑gelatin composite films. In this process, gelatin (2%, w/v) was stirred continuously in distilled water at 80 °C for 30 min to ensure complete dissolution. Thereafter, glycerol was introduced at a concentration of 5 wt% relative to the gelatin mass, serving as a plasticizer. Subsequently, the incorporation of CP (10 wt% based on the dry weight of gelatin) was followed by stirring at 80 °C for 2 h, which yielded a homogeneous solution suitable for film casting, which was then divided into four portions (Lokesh et al., 2022). The control solution was used without further treatment. For ultrasound treatment (US), the film-forming solution (50 mL) was placed in a 100 mL jacketed glass beaker and treated at 200 W and 20 kHz for 30 min using a probe-type ultrasonic processor (JY92-IIN, Ningbo Scientz Biotechnology Co., Ltd., China) equipped with a titanium alloy probe (Φ 6 mm). The probe was immersed to a depth of approximately 1.5 cm below the liquid surface to ensure consistent acoustic field distribution. During treatment, the temperature was kept below 30 °C by circulating ice-water through the jacketed beaker, and the actual temperature was monitored with a thermocouple inserted into the solution. The power of 200 W has been previously optimized for improving the mechanical and barrier properties of biopolymer composite films. The extended treatment time of 30 min was employed to fully promote molecular chain rearrangement and hydrogen bonding reinforcement. The low-temperature condition was maintained to distinguish the mechanical effects of acoustic cavitation from thermal effects (De Vargas et al., 2022). For ultraviolet treatment (UV), the film-forming solution (50 mL) was placed in a quartz vessel (inner diameter 6 cm, height 8 cm) and exposed to UV-C irradiation using a low-pressure mercury lamp (30 W, G30T8, Philips, Netherlands) emitting predominantly at 254 nm. The quartz vessel was positioned directly beneath the lamp at a fixed distance of 15 cm, and the solution was continuously stirred with a magnetic stirrer at 300 rpm to ensure uniform irradiation. The irradiation was performed in a closed UV chamber to prevent external light interference, and the temperature was monitored but not actively controlled, remaining below 35 °C throughout the 30 min treatment. The wavelength of 254 nm has been widely documented to effectively induce crosslinking of gelatin and collagen-based materials through photo-induced covalent bond formation. The treatment duration of 30 min was selected to ensure sufficient crosslinking density on the film surface (M et al., 2014). For microwave treatment (MW), the film-forming solution (50 mL) was placed in a 250 mL round-bottom flask and irradiated in a domestic microwave oven (M1-L23B, Midea Group, China) operating at 2450 MHz with a maximum output power of 300 W. The solution was subjected to intermittent heating: 20 s of irradiation followed by 2 min of cooling (with the flask removed from the oven and placed in a water bath at 25 °C), repeated three times, giving a total effective heating time of 1 min. The temperature was monitored immediately after each heating cycle using an infrared thermometer and maintained below 50 °C. Microwave irradiation at 2450 MHz enables rapid volumetric heating through dipole reorientation, facilitating polymer chain mobility and localized crystallization. The intermittent heating mode was applied to prevent excessive thermal accumulation and protein denaturation (Beikzadeh et al., 2020).

After each physical treatment, 20 mL of the treated film-forming solution was cast onto a leveled polytetrafluoroethylene (PTFE)-coated plate (9 cm × 9 cm) and dried in a forced-air oven at 45 °C for 24 h. After being peeled off, the resulting films were placed in a conditioned environment at 25 °C and 50% relative humidity for 48 h before characterization. The four groups were labeled as Control, US, MW, and UV.

3. **Structural characterization of films**

**3.1. Morphological characterization**

A LEO 1530 VP high‑resolution field‑emission scanning electron microscope (SEM; Carl Zeiss, Oberkochen, Germany) was employed to examine the surface morphology of CP‑gelatin composite films subjected to various physical treatments (Poluri et al., 2026). Before imaging, each sample was coated with a thin gold layer via sputtering for 15 s to enhance electrical conductivity and image resolution. SEM images were acquired at accelerating voltages of 5 or 10 kV and magnifications of 10×, 100×, 500×, 1000×, and 5000×. To evaluate structural uniformity, micrographs were captured from several distinct areas on each film. Surface topography was further evaluated using atomic force microscopy (AFM; Bruker Dimension Icon) (O. et al., 2022). Film specimens were mounted on glass slides, and images were obtained in tapping mode over a scan area of 4.0 μm × 4.0 μm.

**3.2. Fourier-transform infrared (FTIR) spectroscopy**

The structural properties of CP‑gelatin films were characterized by Fourier‑transform infrared (FTIR) spectroscopy using a Nicolet iS20 instrument (Thermo Fisher Scientific, USA). This system was equipped with a deuterated triglycine sulfate detector and a horizontal attenuated total reflectance (ATR) unit containing a germanium crystal. Infrared spectra were acquired over the 4000–500 cm⁻¹ range with a resolution of 4 cm⁻¹, and 64 scans were averaged for both the sample and the background. To confirm reproducibility, a minimum of three independent measurements were performed on different spots of each film. The ATR crystal was cleaned with methanol between analyses. FTIR spectra were used to identify functional groups and evaluate intermolecular interactions, particularly hydrogen bonding, within the film matrix (Stepnov et al., 2024).

**3.3. X‑ray Photoelectron Spectroscopy (XPS)**

The surface elemental composition and chemical states of CP‑gelatin films following different physical treatments were determined using X‑ray photoelectron spectroscopy (XPS). A Thermo Scientific K‑Alpha spectrometer (USA), equipped with a monochromatic Al Kα source (hν = 1486.6 eV), was utilized. The X‑ray source parameters were 12 kV and 6 mA, and the beam diameter was 400 μm. The pressure in the analysis chamber was maintained below 5.0 × 10⁻⁷ mbar. Survey spectra were recorded with a pass energy of 150 eV and a step size of 1 eV, whereas high-resolution spectra of C 1s, N 1s, and O 1s were collected with a pass energy of 50 eV and a step size of 0.1 eV, using at least five signal accumulations. All binding energies were calibrated against the C 1s peak at 284.80 eV. Film samples were cut into approximately 5 mm × 5 mm pieces, mounted on the sample holder, and introduced into the analysis chamber after the pressure decreased below 2.0 × 10⁻⁷ mbar (Ajahar et al., 2023).

**3.4. X-ray diffraction (XRD)**

Wide‑angle X‑ray scattering patterns of CP‑gelatin composite films subjected to different physical treatments were recorded on a Rigaku Miniflex 600 diffractometer (Japan) equipped with a Cu Kα X‑ray tube (λ = 0.15406 nm). The generator was set to 40 kV and 15 mA to characterize the crystalline structure and molecular arrangement of the films (Chen et al., 2024). Film samples were directly placed on a standard sample holder, and diffraction patterns were recorded over a 2θ range of 5°–90° at a scanning rate of 2°/min. The resulting diffraction patterns were used to evaluate the crystallinity and short-range ordered structures of the biopolymer matrix, as well as to assess the effects of ultrasound, ultraviolet, and microwave treatments on intermolecular packing and the potential formation of crystalline domains.

4. **Thermogravimetric Analysis and Differential scanning calorimetry**

Thermogravimetric analysis (TGA) and differential scanning calorimetry (DSC) were employed to assess the thermal behavior of the CP‑gelatin films. (Lu et al., 2025). TGA measurements were performed using a Netzsch STA 2500 thermal analyzer (Germany) under a nitrogen atmosphere. Approximately 8 mg of sample was heated from 30 °C to 800 °C at a rate of 10 °C/min, and weight loss was continuously recorded.

DSC analysis was conducted using a TA Instruments DSC 2500 (New Castle, DE, USA) with a nitrogen flow rate of 50 mL/min. Approximately 5 mg of film was sealed in an aluminum pan and subjected to temperature-modulated DSC (TMDSC) at a heating rate of 10 °C/min, with a modulation period of 60 s and an amplitude of 0.318°C over the temperature range of −40 °C to 400 °C. Temperature and heat‑flow calibration of the instrument was performed with indium, whereas aluminum and sapphire served as reference materials for heat capacity. The resulting TGA and DSC data were used to evaluate thermal stability, decomposition behavior, enthalpy changes, and phase transitions of the films.

5. **Physicochemical and mechanical properties of the films**

**5.1 Thickness and mechanical properties**

The thickness of the conditioned CP-gelatin films was measured at three random locations using a digital micrometer, and the average value was recorded in millimetres (mm). According to ASTM D882 (Nagoor et al., 2023), the mechanical performance of the films, i.e., tensile strength (TS) and elongation at break (EB), was characterized using a universal testing machine (Model 5967, Instron, Canton, MA, USA) under ambient environmental conditions (roughly 22 °C and 60% relative humidity). Film strips (5 cm × 1 cm) were fixed between the grips with an initial separation distance of 30 mm and tested at a crosshead speed of 5 mm/min.

**5.2 Moisture content and water solubility**

The moisture content (MC) and water solubility (WS) of the films were determined according to a previously reported method (Nitong et al., 2023). Briefly, the films were dried at 105 °C for 24 h, immersed in distilled water for 24 h, and subsequently re-dried at 105 °C for an additional 24 h. Following each procedure, the film weight was recorded. Equations (1) and (2) were then applied to calculate the MC and WS, respectively.

(1)MC (%) =$\text{ }\frac{\text{Wi - Wd}}{\text{Wi}}$ × 100

(2)MS (%) =$\text{ }\frac{\text{Wi - Wt}}{\text{Wi}}$ × 100

Where Wi = initial film weight (as‑prepared), Wd = weight after first drying, and Wt = weight after water absorption and re‑drying.

**5.3 Swelling index, water vapor permeability and water contact angle**

The swelling behaviour of the films was evaluated by immersing samples in distilled water at room temperature for 12 h. The swelling index (weight gain, %) was calculated as (Ws − Wd)/Wd × 100, where Ws and Wd represent the swollen and dry weights of the sample, respectively.

Water-vapour permeability (WVP) was determined according to our previously reported method (Priyadarshi et al., 2021). The assembled permeation cups (with the films sealed on top) were weighed at the beginning and then placed inside a conditioning chamber at 25 °C and 50% RH. The weight of the testing samples was measured every 1 h for an interval of 9 h, and a plot was obtained against the weight change of the cup versus time, where the slope displayed the water vapor transmission rate (WVTR, g.m^2^.s) of the films. The WVP (gm/m2.Pa.s) values were calculated as follows:

$$\text{WVP=}\frac{\left( \text{WVTR×L} \right)}{\text{∆p}}\text{ }$$

where L is the mean thickness (m), and Δp is the partial water vapor pressure difference (Pa) of the film.

A JY‑82C video contact‑angle analyzer (Chengde Dingsheng Testing Machine Co., Ltd., Hebei, China) was used to measure the surface hydrophobicity of the prepared films. Film specimens (2.5 cm × 2.5 cm) were fixed horizontally on a black Teflon-coated steel platform. Using a microsyringe, a 10 μL drop of distilled water was gently placed on the film surface. Once the drop became stable, the water contact angle (WCA) was measured (Wang et al., 2025).

6. **Optical properties**

The colour parameters of the films were determined using a Konica Minolta CR‑400 colourimeter (Tokyo, Japan). A white reference plate (L* = 97.75, a* = −0.49, b* = 1.96) was utilized as the background calibration standard. The lightness (L*) and chromaticity parameters, including redness/greenness (a*) and yellowness/blueness (b*), were determined according to (Kim et al., 2024). The total colour difference (ΔE) was calculated as follows:

$$\text{∆E=}\sqrt{\text{(}\text{∆L)}^{\text{2}}\text{+(}\text{∆a)}^{\text{2}}\text{}\text{+(}\text{∆b)}^{\text{2}}}$$

where ∆L, ∆a, and ∆b illustrates the difference between the background color value and the film samples.

The UV–Vis transmittance of the films was measured over the wavelength range of 200–800 nm using a UV–Vis spectrophotometer (Mecasys Optizen POP Series UV/Vis, Seoul, Korea). UV-barrier properties in the UV-A (320–400 nm), UV-B (280–320 nm), and UV-C (200–280 nm) regions were evaluated following the method of (Koutchma et al., 2016). The UV-A and UV-B blocking efficiencies were calculated using Eqs. (1) and (2), respectively.

(1) $\text{UV-A blocking}\left( \text{\%} \right)\text{=100-}\frac{\int_{\text{320}}^{\text{400}} \text{T}\left( \text{λ} \right)\text{dλ}}{\int_{\text{320}}^{\text{400}} \text{dλ}}$
(2) $\text{UV-B blocking}\left( \text{\%} \right)\text{=100-}\frac{\int_{\text{280}}^{\text{320}} \text{T}\left( \text{λ} \right)\text{dλ}}{\int_{\text{280}}^{\text{320}} \text{dλ}}$

With T(λ) being the wavelength‑dependent average transmittance of the film and dλ the spectral bandwidth.

**Fig. S1** Representative force-displacement curves of the CP-gelatin composite

films under different physical treatments.

Ajahar K, Parya E, Jong-Whan R. Chitosan/gelatin-based multifunctional film integrated with green tea carbon dots to extend the shelf life of pork %J Food Packaging and Shelf Life. 37 (2023)

Beikzadeh S, Ghorbani M, Shahbazi N, Izadi F, Pilevar Z, Mortazavian AM. The Effects of Novel Thermal and Nonthermal Technologies on the Properties of Edible Food Packaging %J Food Engineering Reviews. 12: 1-13 (2020)

Chen L, Ke W, Fayong L, Dong X. Green fabrication, characterization and antimicrobial activities of AgO/Ag/carboxymethyl chitosan- graphene oxide films %J Arabian Journal of Chemistry. 17 (2024)

De Vargas VH, Flôres SH, Mercali GD, Marczak LDF. Effect of OHMIC heating and ultrasound on functional properties of biodegradable gelatin-based films. Polymer Engineering and Science 62: 1890-1906 (2022)

Fangfang Y, Jinrui Z, Jiaxin X, Tengfei N, Xin L, Manshun L. Effects of monosaccharide composition on quantitative analysis of total sugar content by phenol-sulfuric acid method&#13; %J Frontiers in Nutrition. 9: 963318-963318 (2022)

Kim SH, Kang SE, Kim YD, Park MK. Industrial-scale blown active packaging film with essential oils: Properties, dual-functional performance, and box packaging application of instant noodles %J Food Packaging and Shelf Life. 43: 101276- (2024)

Koutchma T, Popović V, Ros-Polski V, Popielarz A. Effects of Ultraviolet Light and High-Pressure Processing on Quality and Health-Related Constituents of Fresh Juice Products. Compr Rev Food Sci Food Saf 15: 844-867 (2016)

Li F, Jian Z, Rui Y, Tao A. Extraction, purification, structural features and biological activities of the Opuntia dillenii polysaccharides: A review. %J International journal of biological macromolecules. 314: 144253 (2025)

Lokesh K, Kumar DR, K GK. Antimicrobial packaging film from cactus (Cylindropuntia fulgida) mucilage and gelatine. %J International journal of biological macromolecules. 215: 596-605 (2022)

Lu SY, Zhang JX, Xi M, Li N, Wang ZY. 3D Printable Plasmonic Titanium Nitride Nanoparticles Enhanced Thermoplastic Polyurethane Composite for Photothermal Deicing and Infrared Imaging. Acs Applied Nano Materials 8: 2500-2511 (2025)

M ME, K KC, T TT, D EAK, A YB. Increasing Thermal Stability of Gelatin by UV-Induced Cross-Linking with Glucose. %J International journal of biomaterials. 2014: 979636 (2014)

Nagoor GAA, Juan LS, Zaini MMM, Nor'Izzah Z, Huzaifah MRM, Hasnida RHR, Kavita P, Raza SM, Mahboob A, Mohd R. The Use of Essential Oil Embedded in Polylactic Acid/Chitosan-Based Film for Mango Post-Harvest Application against Pathogenic Fungi. %J Polymers. 15 (2023)

Nitong B, Ning Z, Guoyu C, Ruojun M, Jie P, Chen M, Lin W. Konjac glucomannan/carboxymethyl chitosan film embedding gliadin/casein nanoparticles for grape preservation. %J International journal of biological macromolecules. 249: 126131-126131 (2023)

O. MA, R. DS, I. KA, A. ZS, A. KM, S. ZO, T. GA, F. ZY. Supramolecular Structure and Mechanical Performance of κ-Carrageenan–Gelatin Gel %J Polymers. 14: 4347-4347 (2022)

Pérez M, Dominguez-López I, Lamuela-Raventós RM. The Chemistry Behind the Folin–Ciocalteu Method for the Estimation of (Poly)phenol Content in Food: Total Phenolic Intake in a Mediterranean Dietary Pattern. Journal of Agricultural and Food Chemistry 71: 17543-17553 (2023)

Poluri N, Hao Q, Hu X. Green ultrasonic engineering of hierarchical microphase structures and enhanced properties in chitosan-silk blends across molecular weight ranges. %J Ultrasonics sonochemistry. 126: 107757 (2026)

Priyadarshi R, Riahi Z, Rhim J-W, Han S, Lee S-G. Sulfur Quantum Dots as Fillers in Gelatin/Agar-Based Functional Food Packaging Films. ACS Applied Nano Materials 4: 14292-14302 (2021)

Stepnov AA, Tavera EL, Klauer R, Lincoln CL, Chowreddy RR, Beckham GT, Eijsink VGH, Solomon K, Blenner M, Kolstad GV. Revisiting the activity of two poly(vinyl chloride)- and polyethylene-degrading enzymes %J Nature Communications. 15: 8501-8501 (2024)

Wang YL, Li R, Yang X, Li L. Multifunctional film of soy protein nanofiber/chitosan/zinc oxide for tomato postharvest freshness preservation %J Food Hydrocolloids. 168: 111504-111504 (2025)
